# Notes and News

**Published:** 1889-03-02

**Authors:** 


### Notes and News.

Collected and Communicated.

The Haywood Charity, Burslem, does much for the sick of that neighbourhood. It has a hospital which last year treated 146 in-patients and 3,000 out-patients; besides this the nurses have visited and dressed a large number of out-cases. The expenditure last year was £758, against an income of over £900.

Miss Flavin, a young Catholic lady of Liverpool, has reached New York on her way to Molokai. She is going to take charge of the orphaned lepers of that settlement, and calmly faces the contagion which probably awaits her. Certainly Father Damien's example has had a wide and vast influence.

There is still room for seventeen additional beds at the Royal Portsmouth, Portsea, and Gosport Hospital, if increased subscribers would come forward. The 30 beds in the lock wards are constantly occupied; during last year 130 cases were treated, and owing to benevolent work there carried on, many of the unfortunate patients were persuaded to enter upon a better mode of life.

Dr. J. Brooke Ridley, who has for three years held the position of resident house surgeon at the Hunts County Hospital, has received the appointment (under the Metropolitan Asylums Board of London) of assistant medical officer at their large schools for the training of idiot children at Darenth, Kent. Dr. Brooke Ridley commences his duties at Darenth at the end of the present month.

The Manchester and Salford Provident Dispensaries are doing good work. The increase in the number of members during the past year has been 1,400; there are now 19,000 subscribing members; there are nine dispensaries in association, of which only two are not self-supporting, and the total income for the past year has been over £4,000. There was one remarkable feature in the report, and that was that the percentage of applicants for medical relief at the hospitals which were found on investigation to be ineligible, and which in 1875 was no less than 42 per cent., was last year reduced to the singularly small figure of 6 per cent. Gratuitous medical treatment is therefore not given to the unworthy of Manchester on nearly such a large scale as in London.

A gathering of past and present members of the Board of Management of Bradford Infirmary took place on February 15th, for the purpose of presenting an address to Major Müller on his retirement from the House Committee, after thirty-five years' service. The address was in book form, neatly bound in scarlet Russia leather, and endorsed outside in gold letters, "To Major Müller, on his retirement from the House Committee, Bradford Infirmary." The text was beautifully illuminated and ornamented, and on the same page were contained views of the Infirmary in 1855, 1864, 1874, and 1888, showing the various changes which have been made in the structure since the first-named date, along with a portrait of Major Müller in the left-hand corner.

At the late meeting of the Bradford Infirmary subscribers, the secretary, Mr. W. Maw, read an excellent report. The numbers of cases dealt with during the year were as follow: In-patients, 1,961; home-patients, 2,366; out-patients, 6,153; casualties, 1,954; dental cases, 79; teeth extracted, 281. A library of over 1,000 volumes has been established at the infirmary by the Samaritan Society. The workmen of the town take the greatest interest in this institution, and last year contributed nearly two thousand pounds to its funds.

After the delivery of the Hunterian lecture last week, the president of the College of Surgeons, and his colleagues, entertained a distinguished number of guests in the library of the college. There were present: The Lord Mayor, Sir Andrew Clark, Sir Spencer Wells, Canon Elwyn, Mr. Calderon, the Lord Chancellor, Mr. Ouless, and many more of the notabilities of London society. The evening was a very pleasant one, and the speeches were amusing, Sir Andrew Clark in support of science being specially fine.

The scheme for starting a hospital in the Isle of Skye should be submitted to Mrs. Bishop, *née* Bird. Doubtless many of our readers remember that this energetic lady, before marrying Dr. Bishop and settling in Mull, went through a short course of nursing at St. Mary's Hospital. She then started a small hospital close to her home in the Western Highlands, but, if report speaks true, the hospital is not a success. The Highlanders are an independent folk, and it is to be hoped that charity will only be offered them by those who understand their character and their needs.

The supporters of Salford Royal Hospital had one or two differences of opinion at their last meeting. To begin with, the medical staff wished that all surgeons of the institution should be Fellows of their college, while Mr. Stocks and others held that Fellows were not necessarily better operators than members, and that members should be eligible for such posts. The lay party carried the day, upon which the medical party withdrew in disgust. The system of "recommends" was then discussed, but the majority were in favour of this old-fashioned system.

A more favourable report of the Essex and Colchester Hospital reaches us than we have seen of late years. There is still a small balance due to the treasurer, but there seems some hope that another twelve months will see the balance on the other side. One-third of the net income arises from the Hospital Saturday and Sunday Funds, which show an increase of £46 over last year. The patients have been very numerous lately, and the staff has had plenty of work. The report acknowledges this, and closes with an expression of thanks to all, especially the house surgeon, matron, and secretary.

The first report of the Wimborne Jubilee Hospital is out, and gives an interesting account of the start of this small institution. Immediately after the appointment of the committee at the commencement of last year they met and began the work of framing rules. This took a considerable time, but the task was accomplished, and on the whole the rules work well. The committee having meanwhile advertised for a nurse-matron, a large number of replies were received. Eventually, Miss Mary Davidson, late of the Royal Infirmary, Edinburgh, was selected. She entered upon her duties early in March. On March 5th the hospital was opened for patients, and before many days two or three of its beds were filled. Since then there has been no lack of patients. During the ten months it has been open there have been received 34 cases. In November Miss Davidson resigned, and was succeeded by Miss Baker. The committee still have a balance of £230 left over from the Jubilee fund; £15 of this is to be expended in building a porch.

**THE following vacancies are announced this week, the amount of salary in each case being given after the name of the institution :—**

**Resident Medical Officers.**

Birmingham General Hospital. Medical Officer.—£130, board, etc.
Liverpool Northern Hospital. Assistant House Surgeon.—£70, board, etc.
Hemel Hempstead Infirmary. House Surgeon and Dispenser.—£100, board, etc.
Victoria Hospital for Children, Chelsea. House Physician.—£50, board, etc.
Rotherham Hospital. Assistant House Surgeon.—Board, etc.
Jessop Hospital for Women, Sheffield. House Surgeon.—£50, board, etc.

**Non-resident Medical Officers.**

Manchester Owens College. Lecturer on Skin Diseases.
King's College, London. Professor of Forensic Medicine.
London Fever Hospital, Islington. Physician and Assistant Physician.
Guy's Hospital. Six Dental Surgeons, Tutor, etc.
Nottingham Friendly Societies. Assistant Medical Officer.—£120.
West End Hospital, Welbeck Street. Physician and Ophthalmic Surgeon.

---

A GENTLEMAN has offered to contribute £1,000 to the Samaritan Society, provided another £4,000 can be raised, the whole sum to be invested, and the interest applied to the needs of the charity. The work consists in supplying patients with mechanical aids, such as artificial arms, legs, etc., and sending those who are convalescent to the seaside or country. The number of patients relieved last year was 3,999. The secretary is Mr. Thomas Hornsby, Samaritan Society, London Hospital, Whitechapel.

---

BELFAST people are specially liable to consumption, partly owing to the nature of the industries of that town ; they have, therefore a special hospital for consumption. The record of this "Throne Hospital" for the past twelve months shows that out of thirty-three patients under treatment during that period, nine were discharged in a much-improved condition. In other words, the skill and attention bestowed on the patients in the institution resulted in 27·25 per cent. being sent forth with renewed strength and vigour to face the world. But the report was not all pleasant reading. It told the old tale of numerous applicants for admission having to be sent away for want of accommodation. So urgent have the demands upon the resources of the Hospital become that the committee have, with commendable energy, provided room for two additional beds. It is estimated that the expense of each of these beds would be about £80 a year. Mr. Murray nobly offered £100 towards the endowment of one of these beds ; and it is hoped his example may be followed.

---

THE oldest Cabinet Minister is Viscount Cranbrook, G.C.S.I., Lord President of the Council, aged 74 ; the youngest is the Right Hon. Arthur James Balfour, M.P., Chief Secretary for Ireland, aged 40. The oldest member of Her Majesty's Privy Council is the Right Hon. Lord Cottesloe, aged 90 ; the youngest, the Duke of Portland, aged 31. The oldest duke is the Duke of Cleveland, K.G., aged 85 ; the youngest, H.R.H. the Duke of Albany, aged 4. The oldest prelate of the Church of England is the Right Rev. Richard Durnford, D.D., Bishop of Chichester, aged 86 ; the youngest, the Right Rev. Francis John Jayne, D.D., Bishop Designate of Chester, aged 44. The oldest prelate of the Irish Episcopal Church is the Most Rev. Robert Bent Knox, D.D., Archbishop of Armagh, aged 81 ; the youngest, the Right Rev. Robert Samuel Gregg, D.D., Bishop of Cork, Cloyne, and Ross, aged 54. The oldest knight is Sir Provo William Parry Wallis, G.C.B., the Senior Admiral of the Fleet, of Funtington House, near Chichester, aged 97 ; the youngest, Sir Edgar Vincent, K.C.M.G., Financial Adviser to the Khedive of Egypt, aged 31.—*Who's Who in* 1889.

AT the annual meeting of the Croydon Provident Dispensary members, Dr. Carpenter made a capital speech, in which he pointed out the value of co-operation. There was no mistake, he said, that the dispensary could never be as efficient as it ought to be until they had two or three thousand members. It was a co-operative movement, by which a great number of small sums made a large amount, and in a co-operative movement they ought to support one another. That was the great principle that underlaid all dispensaries, there was a thought for each other. The more they co-operated the more advantageous would their institution become. It was a movement which tended to make Englishmen independent and thrifty, and enable them to protect themselves and their own. Every young man ought to be a member of a dispensary, and thus protect himself from those sudden influences which tended to bring pauperism on those who never intended to be paupers. Some sudden event reduced them from a condition of comfort to one of indigence, and after a short struggle they were compelled to go into the workhouse, and many there were in the workhouse at the present time whose presence there was owing to their having neglected to observe these precautions in their youth.

---

THE President of Dewsbury Hospital enlivened the annual meeting by giving some particulars of last year's cases, which he had procured from Mr. Milne and Mrs. Neilson. From Ossett came a youth of sixteen with both thighs and the left leg broken while at work in a factory. He was an inmate of the infirmary for about three months, and left completely cured. From Liversedge there had been some serious surgical and medical cases. In the first was a young man, who, while working in a coal pit there, was terribly injured by a fall of roof. He was an in-patient for forty-six weeks, and his condition was such, being paralysed from the waist downwards, that his bedclothes required to be changed thrice and oftener daily, and his position in bed altered almost as often each hour. That sufferer was removed to an incurable hospital at Harrogate, and it said much for the careful nursing he had received that there was not a single bedsore upon him. A Mirfield miner similarly, though not so dreadfully, injured was cured in ten weeks, and enabled to again earn his living. Among the medical cases was a girl of thirteen years, belonging to Dewsbury, who on admission only weighed two stones, but who gained one stone five pounds while in the infirmary, and was able to leave cured. These stories, of course, interested the hearers much more than the usual dry list of figures.

---

AT the late annual meeting of Cork Fever Hospital subscribers, Dr. Moriarty read a long and interesting report of the medical work done at the hospital. The total number of cases, he said, treated during the year 1887 was 173, and for the present year, 154. The mortality from all causes has been at the rate of 10 per cent., and when we deduct the cases hopeless on admission, the death-rate is 6 per cent., a result which will favourably compare with the good work of any previous year. In the early part of the year measles of a bad type prevailed as an epidemic. The disease was so general that almost all those of early life suffered from it. In the extreme south of this county the disease was very fatal, but a great part of the mortality was apparently due to bad living and bad hygienic surroundings. Do what we will our people will not advance with the progress of sanitary legislation, neither will they care to change their old ways of living until such time as they are coerced to do so by fines and imprisonment. Dr. Moriarty then referred to the fact that typhus was still with them, and that enteric fever was also general. In speaking of the last he told how the soldiers of Fermoy barracks sickened after eating diseased meat supplied by contractors, and said that typhoid at Dublin barracks was largely due to impure milk. Dr. Moriarty told some stories of how scarlatina is spread by carelessness, and concluded with some sensible remarks on the prevention of disease.

